# A Setup for Camera-Based Detection of Simulated Pathological States Using a Neonatal Phantom

**DOI:** 10.3390/s22030957

**Published:** 2022-01-26

**Authors:** Florian Voss, Simon Lyra, Daniel Blase, Steffen Leonhardt, Markus Lüken

**Affiliations:** Chair for Medical Information Technology, Helmholtz Institute for Biomedical Engineering, RWTH Aachen University, 52074 Aachen, Germany; lyra@hia.rwth-aachen.de (S.L.); blase@hia.rwth-aachen.de (D.B.); leonhardt@hia.rwth-aachen.de (S.L.); lueken@hia.rwth-aachen.de (M.L.)

**Keywords:** hardware phantom, neonatal care, NICU, PPG simulation, thermoregulation, vital signs measurement

## Abstract

Premature infants are among the most vulnerable patients in a hospital. Due to numerous complications associated with immaturity, a continuous monitoring of vital signs with a high sensitivity and accuracy is required. Today, wired sensors are attached to the patient’s skin. However, adhesive electrodes can be potentially harmful as they can damage the very thin immature skin. Although unobtrusive monitoring systems using cameras show the potential to replace cable-based techniques, advanced image processing algorithms are data-driven and, therefore, need much data to be trained. Due to the low availability of public neonatal image data, a patient phantom could help to implement algorithms for the robust extraction of vital signs from video recordings. In this work, a camera-based system is presented and validated using a neonatal phantom, which enabled a simulation of common neonatal pathologies such as hypo-/hyperthermia and brady-/tachycardia. The implemented algorithm was able to continuously measure and analyze the heart rate via photoplethysmography imaging with a mean absolute error of 0.91 bpm, as well as the distribution of a neonate’s skin temperature with a mean absolute error of less than 0.55 °C. For accurate measurements, a temperature gain offset correction on the registered image from two infrared thermography cameras was performed. A deep learning-based keypoint detector was applied for temperature mapping and guidance for the feature extraction. The presented setup successfully detected several levels of hypo- and hyperthermia, an increased central-peripheral temperature difference, tachycardia and bradycardia.

## 1. Introduction

Worldwide, about 10% of all children are born prematurely, which results in 15 million premature infants each year. Even though advanced medical treatment in combination with technical development have already led to a 50% reduction in child mortality from 1990 to 2019 [1,2], the World Health Organisation (WHO) estimated 2.4 million deaths in 2019, which means on average about 6700 neonates per day worldwide [3]. About one million of these infants die due to complications related to preterm birth, making prematurity the second-leading cause of death in children under 5 years in 2012 [4].

Despite the high mortality, only data of 2.5% of global neonatal deaths are based on reliable vital registration systems, while the rest originates from estimations and surveys [5]. Prematurity is an often occurring phenomenon with rising rates. In 2012, the WHO reported that 5–18% of all infants were born preterm. The mortality of these patients strongly decreases with higher gestational age, higher birth weight, and more expensive care. In a neonatal intensive care unit (NICU), the special care especially focuses on keeping the newborns’ body core temperature within a range, which maximizes their chances of survival and is beneficial regarding morbidity and growth [5]. This support is vital, because the thermoregulation of a preterm neonate cannot yet handle large deviations of body temperature, which could lead to a deterioration of the health state due to hypothermia or even death from associated severe infections [6]. Regardless of the core temperature, changes in the central-peripheral temperature difference can indicate abnormal clinical states, such as an unspecific sign of a sepsis [7].

Preterm infants receive medical care inside an incubator in a NICU where several vital signs such as heart rate (HR), respiration rate, oxygen saturation and temperature are monitored. The continuous surveillance of the cardio-respiratory system enables the determination of a patient’s health status and an early detection of a deterioration of this state. Furthermore, monitoring a newborn’s thermal condition can also help to detect signs of abnormal clinical states such as cold or heat stress as well as infections by closely tracking not only the central but also the peripheral temperature [6,8]. While adhesive electrodes are used for the measurement of cardio-respiratory activities, the thermoregulation monitoring is usually conducted using adhering thermistors, whose resistance varies with temperature. Despite benefits such as low price and easy handling, the removal of the probes can cause skin irritations to the still immature skin and induce wounds, which can lead to an infection [9]. Additionally, wired probes require cables, which not only complicate transport and interaction like kangaroo-care, but also cause psychological stress for the parents [10]. To overcome the disadvantages of wired patient-monitoring for neonates, contact-free camera surveillance may be a promising technology and is welcomed by clinical staff [11].

The research interest in the field of camera-based vital signs measurement has continuously increased in recent years. While RGB cameras can be used for Region-of-Interest (ROI) detection and monitoring of vital signs such as HR using photoplethysmography imaging (PPGI) and respiration activity, infrared thermography (IRT) devices can measure the surface temperature of objects and, therefore, are able to derive the thermal state of a patient [12]. Since continuous vital sign monitoring requires real-time data processing, classical approaches from the field of medical imaging may not be applicable due to the high computing power required. In contrast to this, deep learning (DL)-based approaches have shown to enable real-time image processing for clinical surveillance [13,14]. To determine the HR or skin temperature distribution of the neonatal body, segmentation or keypoint detection algorithms can be used, featuring contact-less supervision of all desired and visually accessible body parts [12,15]. These algorithms could further facilitate contactless alarm systems by classifying the extracted vital signs for pathology detection. However, for the training and validation steps of such DL-based algorithms, large datasets with various disease progressions are required, which are not publicly available at this moment. Therefore, the literature in this field is still in an early stage.

So far, there have been several studies about neonatal pathology detection which used vital signs recorded from cable-based sensors in combination with DL or machine learning (ML). In 2019, Ansari et al. [16] published a study about neonatal seizure detection from electroencephalography (EEG) data using deep convolutional neural networks (CNNs). In the same year, Ihlen et al. [17] applied ML on neonatal movement data for the early prediction of cerebral palsy. One year later, Turova et al. [18] applied ML models for the prediction of cerebral hemorrhage in infants from clinical data. In addition, in recent studies DL was used to support vital signs monitoring and pathology classification from images [19]. In 2019, Villarroel et al. [20] implemented a multitask DL algorithm to segment skin areas and estimate vital signs in a NICU. In 2021, Huang et al. [14] trained spatio-temporal neural networks for non-contact neonatal HR monitoring. Furthermore, Nagy et al. and Khanam et al. [19,21] both proposed an algorithm for the measurement of pulse and breathing rate. Simultaneously, Ervural et al. [22] classified neonatal diseases using thermographic data in combination with CNNs. Although these DL-based approaches showed promising results, the models were trained on relatively small neonatal image datasets. Thus, any statement on generalization of the models is difficult. To overcome the issue of missing large neonatal image datasets, one solution could be the generation of artificial data using a neonatal phantom which can simulate vital signs such as cardiac activity and thermoregulatory processes and enables the configuration of pathological states. In a previous work, we introduced a neonatal phantom, which is able to simulate a PPG signal and thermoregulatory processes on the skin surface in individually controllable areas [23].

In this work, we present a low-cost, camera-based monitoring approach which is able to measure vital signs simulated by such a neonatal phantom inside an incubator in order to detect pathology patterns. A pre-trained DL-based keypoint detector was used to extract ROIs on the skin surface of the phantom and monitor vital signs such as HR and thermoregulation in real-time. During live monitoring, pathological vital signs patterns were set for cardiac activity and temperature distribution, which were independently measured and classified with the camera-based system. A simultaneous simulation of vital signs using the neonatal phantom and the camera-based measurement of the signals with a deep learning-supported detection of pathological patterns have so far not been reported in the literature, and are considered a novel contribution.

## 2. Pathology Simulation

### 2.1. Neonatal Phantom

**Figure 1:**
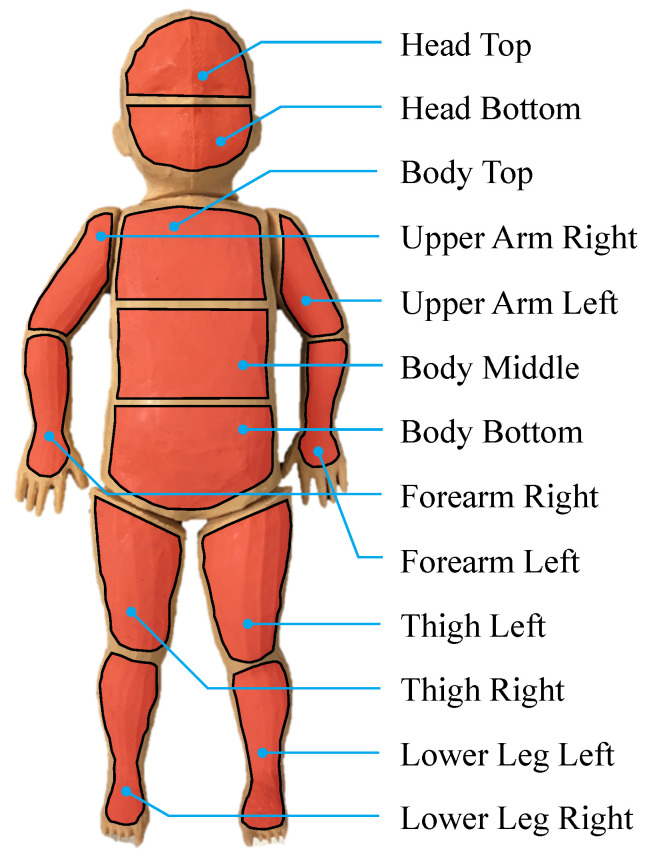
Individual heating regions adapted from [23]. While the head and the limbs were divided into two heatable areas, the torso was subdivided into three regions.

As presented in [23], a 3D-printed scaffold was covered with different layers of silicone to resemble the shape and outer appearance of a premature neonate. It was manufactured using rigid acrylonitrile butadiene styrene (ABS) and equipped with hardware components such as carbon-based heating elements to simulate a temperature distribution on the body surface and LEDs for PPG emulation. Temperature sensors were placed next to the thermal components to enable feedback control. Since these sensors were located in different positions for each region, the measured skin temperature deviated from the corresponding temperature sensor. Thus, an individual offset was added to the set temperature for each region, enabling a stable control of the skin temperature. The neonatal phantom and all individually controllable heating regions can be seen in Figure 1. While the head and the limbs are divided into two regions, the torso can be heated with three individual areas.

An LED-driven PPG emulation enabled the simulation of pulsatile blood flow through the body, distributed over all body parts. In total, 48 red and 60 infrared LEDs were integrated into the scaffold for HR simulation. The wiring of all hardware components was guided to the back of the phantom, where it was connected to the main board holding the ultra-low-power *STM32L4R7VIT* (STMicroelectronics, Switzerland) and the required peripherals to control the simulation [23].

### 2.2. Simulation Routine

**Figure 2:**
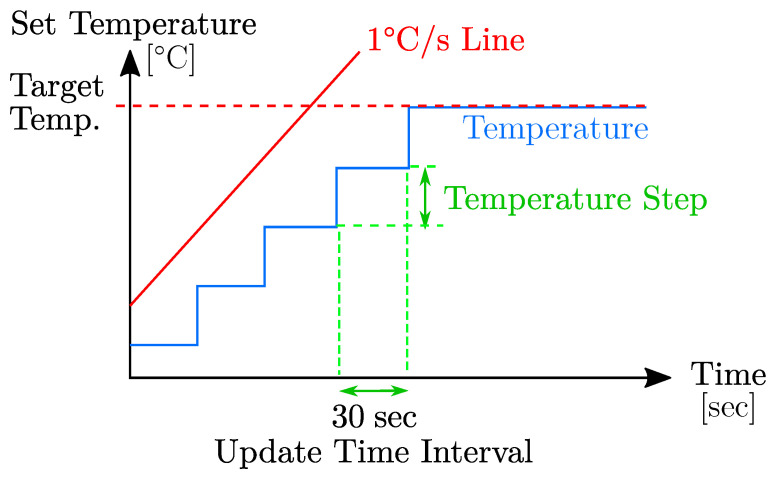
Routine during a temperature increase. The set temperature was adjusted after an update time interval of 30 sec. The routine was applied until the target temperature was reached.

The phantom was connected to a host computer running MATLAB R2020a (MathWorks, USA) to adjust user-defined temperature and HR settings. The operation of the phantom was implemented using profiles for the temperature process and HR behavior. A temperature profile for a certain pathology consisted of a target temperature for every body part, a temperature step width and the current set temperature. Every 30 s, one temperature step width was added to the set temperature, until the body part had reached its target temperature. After that, the set temperature was not updated any further, until the time given for the execution was over, as illustrated in Figure 2.

The temperature steps were implemented to ensure heat-up speeds of less than $1\;{}^{\circ }C$/min, resembling typical temperature dynamics. The value was based on the temperature difference between the first step and the target temperature. A decrease in temperature did not need to be limited by software, because the sufficiently small temperature differences did not lead to unusually fast temperature drops. Hence, the set temperature was directly set to the target temperature without further updates.

The temperature-related pathologies, along with their detection thresholds simulated in this work, can be found in Table 1. In general, varying temperature boundaries are provided in the literature, leading to a persistent disagreement on what is considered as a normal body temperature in newborns. However, most studies and guides follow the WHO guidelines [24,25,26].

The physiological temperature ranges for pathology detection in this work were specified according to the WHO, with the normal range of body temperature being 36.5–$37.5\;{}^{\circ }C$ [27]. While all temperatures above $37.5\;{}^{\circ }C$ are referred to as hyperthermia, the hypothermia is subdivided into three different stages. The range 36–$36.4\;{}^{\circ }C$ indicates a mild hypothermia which should trigger cause for concern. If the body temperature drops below $36\;{}^{\circ }C$, it is defined as moderate hypothermia, which should prompt immediate rewarming of the infant. Temperatures below $32\;{}^{\circ }C$ are classified as severe hypothermia, which can be life-threatening [27]. Additionally, we defined a level of severe hyperthermia for skin temperatures above $40\;{}^{\circ }C$. Regarding changes in the central-peripheral temperature difference, we defined a pathology parameter of a cpTD greater than 2 ${}^{\circ }C$ according to the guideline of the Society for Neonatology and Pediatric Intensive Care Medicine Germany [25].

Additionally, a simulated HR profile can be chosen, which includes the target heart rate $HR_{T}$ in bpm and a fixed HR variability (HRV). The actual HR value $HR_{S}$ (set value) is a cumulative variable, which will be updated every 5 s by adding a pseudorandom integer value *r* with a maximum step width $r_{\max}$, in order to enable a random HR variation. Boundary checks make sure that the HR boundaries $HR_{\mathrm{Lim}}$ are not surpassed. In our work, we assumed the HR boundaries and maximum step width to be $HR_{\mathrm{Lim}}=HR_{T}\pm 10\;\mathrm{bpm}$ and $r_{\max}=10\;\mathrm{bpm}$ for the simulation of a neonate. It should be mentioned that HRV-related pathologies were not in the scope of this work and were thus not further investigated.

The HR-related pathologies along with their detection thresholds simulated in this work are listed in Table 2. The most common indications are *tachycardia* and *bradycardia*. As of today, there is no global agreement for an average HR value defined as highest or lowest normal. We used the threshold values suggested by the *American Academy of Pediatrics* [28] and in the *Heidelberger Guideline for Neonatology* [29].

## 3. Setup Implementation

The presented neonatal phantom was placed inside a closed incubator of type *Thermocare Vita* (Weyer, Germany), which has an infrared (IR) inspection window *ClearIR-4-P* (IRISS, USA) inserted into the hood to allow thermal radiation to pass. The setup should be low-cost, highly accurate, and at the same time compact enough to be placed on top of the incubator.

### 3.1. Hardware Setup

A *Jetson Xavier NX* (NVIDIA, USA) was used as image processing unit for the setup. This embedded GPU is a very powerful yet small system-on-module, which allows running deep neural networks and to process data from multiple camera sensors in real-time. The included 6 Carmel ARM CPUs, 384 CUDA cores, and 48 tensor cores enable a high performance for image processing. The Jetson module was used for image acquisition and processing, as well as ROI detection (cf. Section 3.3).

The hardware setup was implemented using a low-cost multi-modal recording system consisting of two infrared thermography devices and one RGB camera. For thermal imaging, we integrated two long wave infrared (LWIR) cameras of type Lepton 3.5 (FLIR Systems, USA) into the hardware setup. These low-power, uncooled, FPA microthermal imaging modules are easily integratable into small camera system designs. With respect to the distance of the cameras due to the geometry of the incubator, one Lepton was not sufficient to overview the whole mattress area. Therefore, two thermal sensors were arranged in a line, but slightly tilted. The absolute temperature accuracy is given as $\pm 5\;{}^{\circ }C$ and the module includes an on-board shutter for live calibration. Each Lepton has a horizontal field-of-view (HFOV) of 57${}^{\circ }$, which in combination allows to guarantee a large enough FOV.

Further, we used one RGB camera module for ROI determination and PPGI extraction. The *Arducam MINI High HQ Camera Module* (Arducam, China) uses the 12.3 MP SONY IMX477 sensor with an optical format of 1/2.3${}^{\prime \prime }$ and a M12 mount lens, enabling video streaming with up to 4K at 30 fps [30]. The Arducam uses the camera serial interface (CSI) specification for connection and can be connected directly to the Jetson module. The most relevant properties of both cameras are listed in Table 3.

**Figure 3:**
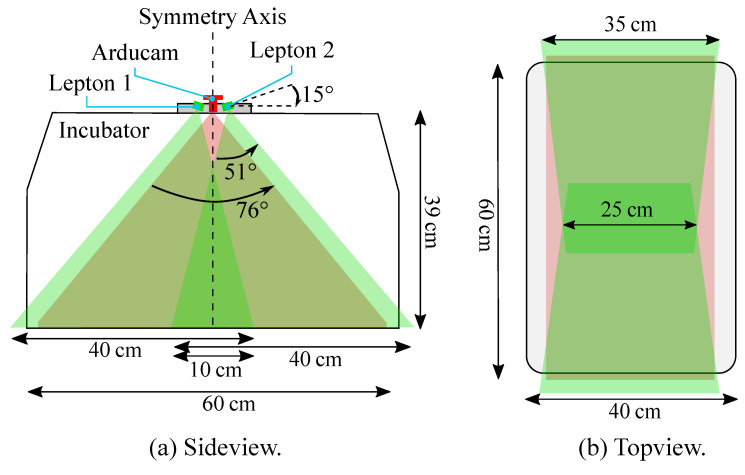
(**a**) Side view of the arranged camera setup and field of view. (**b**) Top view of the arranged camera setup and the covered mattress area.

While a Full-HD resolution was applied for the RGB camera using a frame rate of 30 fps, the IRT device had a slower frame rate of approx. 9 fps (maximum frame rate), which was, however, sufficient for measuring the inert temperature deviations. Since the camera setup is supposed to overview the entire mattress of the incubator, considerations regarding area coverage of the Leptons (in green) and the Arducam (in red) were done as depicted in Figure 3a.

The Lepton cameras were tilted by $15{}^{\circ }$ against the horizontal line, and with their wider viewing angle parallel to the longer axis of the incubator, in order to capture as much of the mattress area as possible. With this arrangement and the usage of two Leptons, the whole scene could be captured, apart from some small cutouts in the overlapping area (cf. Figure 3b). The Arducam was centered in between the Leptons and had a large HFOV, providing an overview of the whole mattress.

A modular housing, holding all three cameras and the Jetson Xavier NX, was designed using the 3D CAD software *Fusion 360* (Autodesk, USA) and 3D-printed using an *Ultimaker 3* printer (Ultimaker, The Netherlands).

### 3.2. Thermal Calibration

Despite their thermal sensitivity of 0.05 K, the Leptons currently have a nominal absolute temperature accuracy of only ±$5\;{}^{\circ }C$ in the high gain mode (range of −10 to 140 ${}^{\circ }C$). Considering the small size and the low price of this thermal imaging sensor, the accuracy is relatively high. Nevertheless, it should be mentioned that it is about as high as the thermal dynamics, which will be simulated during this work. Therefore, a thermal calibration was performed to improve the absolute measurement accuracy. In fact, in combination with the IR window the Leptons were found to overestimate low temperatures and underestimate higher temperatures, especially after applying the Lepton’s calibration shutter. Thus, a linear temperature calibration according to (1) was chosen to convert the measured surface temperature $T_{m}$ to the true surface temperature $T_{s}$. Apart from the slope correction $K_{1}$, an offset $K_{2}$ is assumed.

(1) $$\begin{array}{cc} \; & T_{s}=K_{1}\;\cdot \;T_{m}+K_{2} \end{array}$$

**Figure 4:**
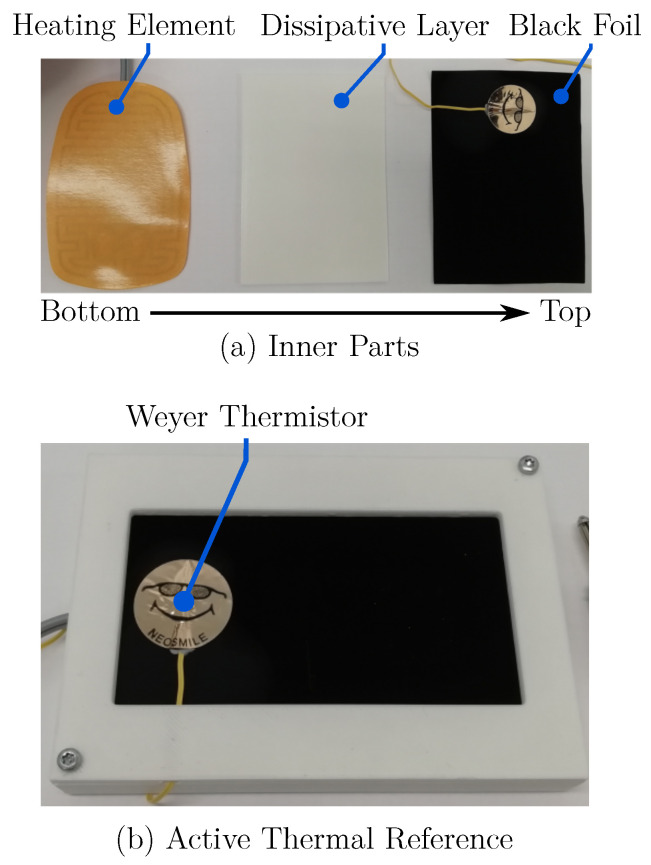
(**a**) Inner parts of the reference device. The heating elements was placed under a dissipative layer and an overlying black foil. (**b**) Assembled active thermal reference body with an attached Weyer thermistor.

In order to determine both the slope correction $K_{1}$ and the offset $K_{2}$ of the temperature calibration, a heatable black body was developed and used as an active thermal reference. Therefore, a heating foil (Thermo, Germany) and a more than 98% emittive, black coated aluminum foil (Advanced Coating, Germany) were integrated into a 3D-printed ABS housing, resisting temperatures of at least $60\;{}^{\circ }C$. Both foils are separated by a thin layer of ABS, which is supposed to cause a more homogeneous temperature distribution in the black foil. To obtain the reference temperature, a Weyer skin temperature probe (Weyer, Germany) was placed on the black foil. These adhesive thermistors are clinically used as the gold standard for neonatal skin temperature measurements and have a range of $0\;{}^{\circ }C$–$50\;{}^{\circ }C$. The maximum error of the probes were specified as ±$0.1\;{}^{\circ }C$ [32]. The active thermal reference body is shown in Figure 4.

### 3.3. Software Implementation

**Figure 5:**
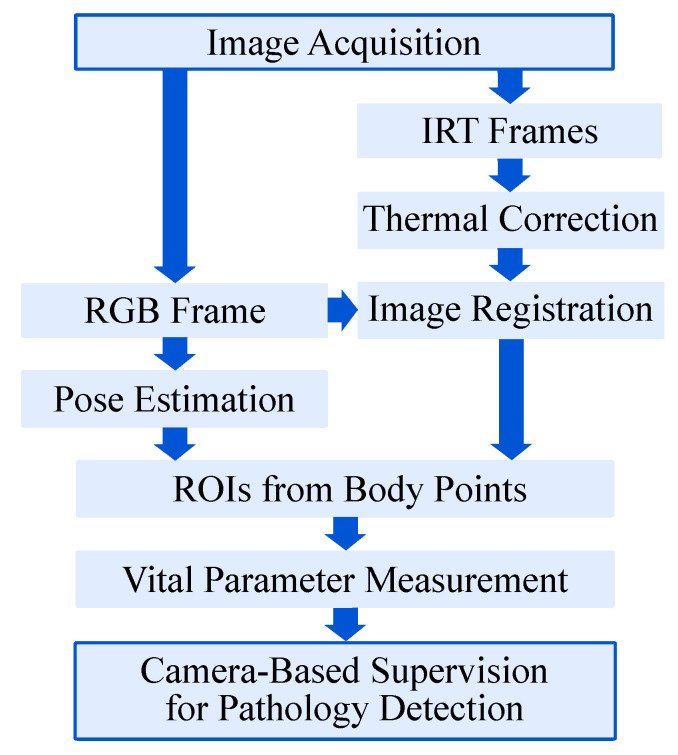
Overview of the pathology detection algorithm. Multithreading was used for parallel data processing.

Since image data from several cameras need to be processed simultaneously, the software routine of the setup was designed as a multithreaded process in Python 3. First, the frames were acquired from the multi-modal camera devices. Subsequently, the gain-offset correction for the IRT frames was performed as presented in Figure 5.

The infrared images and the RGB frame were then registered and transformed, allowing both image modalities to have the same Point-of-View (POV). Afterwards, the RGB frame was used to determine the ROIs using a body pose estimation (BPE) algorithm. Before obtaining the desired vital parameters, the thermal correction on the transformed infrared images was done. Finally, the PPGI signal for HR detection and the temperature values were extracted from the RGB and the IR image, respectively. The single steps of the algorithm are explained in more detail in the following section.

**Figure 6:**
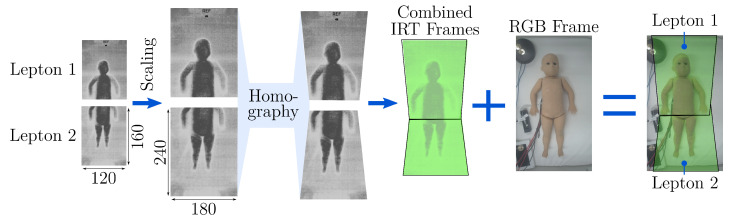
Overview of the registration algorithm.

Regarding the image registration, a manual approach was chosen by selecting ten points on the IRT image and the corresponding feature points on the RGB image. Using the Ransac algorithm [33] for optimal matching, these feature were taken to determine a 3 × 3 homography matrix *H*, which can rotate, translate and distort the Lepton images to match the RGB image. As the Leptons have a fixed position in their housing, the manual labeling process described above was only done once. The registration process is shown in Figure 6. After rescaling of both Lepton frames, they were transformed using the homography matrices $H_{L1}$ and $H_{L2}$ to create the combined IRT image. The overlapping region was filled with temperature data from Lepton 1 that mainly records the upper body.

In order to determine ROIs on hands, feet, the forehead and the chest, we used a state-of-the-art DL-based BPE algorithm. The BPE was executed on the images captured by the RGB camera rather than the IRT camera, because existing open-source models are pre-trained in RGB. During this work, the NVIDIA project *trt_pose* was used, which enables a BPE on embedded NVIDIA Jetson modules [34]. No further transfer-learning step using additional data was conducted, but only the pre-trained version has been used. The network architecture is based on the *CMU-Pose* real-time multi-person 2D pose estimator of Cao et al. [15]. In contrast to *CMU-Pose*, the NVIDIA approach works on feature maps extracted from an image by a ResNet-18 feature extractor instead of the 10 first layers of VGG19, as suggested by Xiao et al. [35].

**Figure 7:**
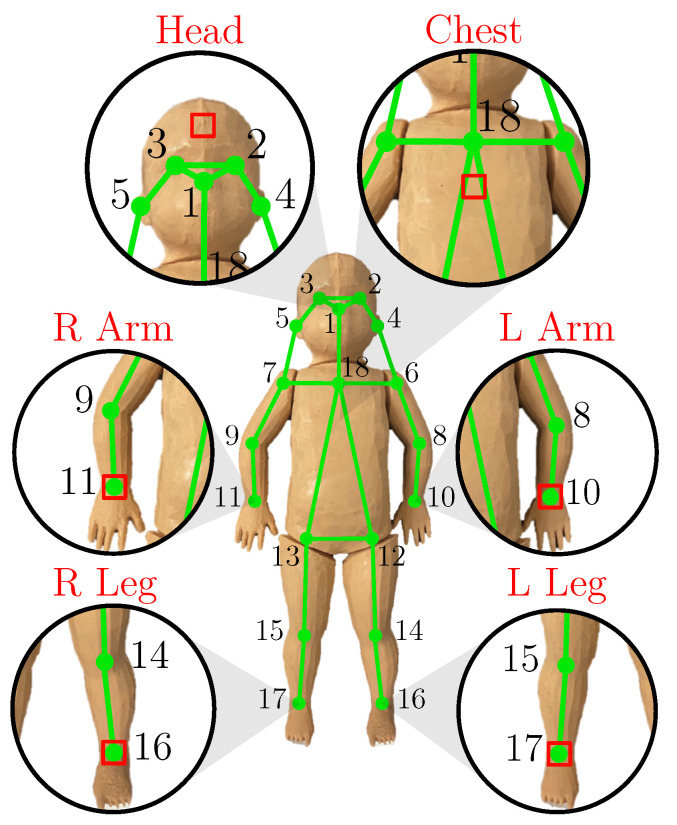
Detected keypoints on the neonatal phantom with extracted ROIs for temperature and HR measurement.

*CMU-Pose* is a two-branch multi-stage CNN, with one branch for learning Part Affinity Fields (PAFs) and one for learning Confidence Maps (CMAPs). A CMAP describes the area, where the respective body part can be found. The PAFs represent a 2D vector field which shows the association between two body points. For example, the body part could be the left forearm, connecting the keypoints in the left elbow and the left wrist. Subsequent stages in the CNN receive the CMAPs and PAFs as well as the initial features F to refine the solution. In contrast to *CMU-Pose*, the *trt_pose* approach optimized the architecture by preventing the feature exchange between the CMAP and PAF branches, which decreased the computational effort. The matching of keypoints with individuals is performed using a greedy graph matching algorithm, where every association gets a weight and the association with the largest weight was selected [15]. An overview of the keypoints delivered from the pose estimation and the desired six ROIs for temperature and HR measurements is illustrated in Figure 7.

With the detected ROIs, the skin temperature distribution of the phantom was obtained from the infrared image. Since red LEDs were integrated into the neonatal phantom, the extraction of the heart rate $HR_{\mathrm{PPGI}}$ was performed by taking the mean intensity of the red channel inside a head ROI for each RGB frame. A *find_peaks* function was applied to detect maxima in the signal. Since small peaks caused by noise or the smaller, local maxima of the simulated LED brightness would lead to a corrupted HR, the maxima were compared to the minimum values to select only major peaks. The time distance between the detected peaks was evaluated and irrelevant HRs above 300 bpm and below 36 bpm were filtered out, as they were considered to originate from noise. The use of $T_{Mean}$ as mean of the time distance array, which contained only valid measurements from the last 10 s, enabled the calculation of the estimated heart rate $HR_{\mathrm{PPGI}}$.

## 4. Results

The validation measurements were divided into the evaluation of the thermal calibration and pathology detection. Furthermore, the results were organized into simulation along with detection of thermal and HR-related pathologies. All experiments were performed inside an incubator Thermocare Vita (Weyer, Germany). The incubator was heated to a constant set temperature of 30 ${}^{\circ }C$. This initial temperature was chosen since it enabled a simulation of thermal pathologies in the range between 30 ${}^{\circ }C$ and 40 ${}^{\circ }C$.

### 4.1. Thermal Validation

The temperature accuracy of both Leptons after temperature correction was examined using an active thermal reference body (cf. Section 3.2). Since the measurement range focused on was 30–$40\;{}^{\circ }C$, only the error (mean of absolute difference) in this band contributed to the thermal calibration and the calculated accuracy.

In the first step, the heating elements were driven with a voltage of around 4 V and a current, which had been slowly increased and decreased between 0.175 A and 0.575 A. Consequently, the active reference reached temperatures between $26\;{}^{\circ }C$ and $45\;{}^{\circ }C$. The Lepton 1 (cf. Section 3.1) was used to measure temperatures and determine the slope correction along with the offset of the thermal calibration. The resulting linear fit is given in (2).

(2) $$\begin{array}{cc} \; & T_{s}=1.82\;\cdot \;T_{m}-20.18 \end{array}$$

**Figure 8:**
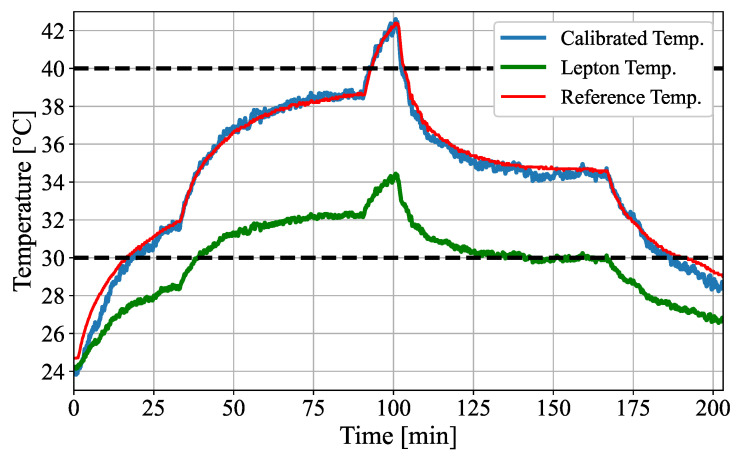
Validation measurement for temperature calibration.

In the second step, the active reference measurement was repeated, using the Lepton 2 to validate the thermal calibration. Figure 8 shows the validation measurement, as well as the calibrated and reference temperature.

The calibrated temperature was well-fitted to the reference temperature, which was recorded using the gold standard temperature probe. Since the thermal calibration was optimized for the temperature range of 30–40 ${}^{\circ }C$, calibrated temperatures below or above this range deviated more from the reference. In addition to the temperature curves in Figure 8, the mean absolute error (MAE), the standard deviation (STD) as well as maximum (MAX) and minimum (MIN) error of both measurements (Lepton 1 for fitting and Lepton 2 for validation) are presented in Table 4.

The comparison between the calibrated temperatures of Lepton 1 and Lepton 2 revealed that the validation measurement had even smaller MAE, STD, MIN and MAX error than the measurement used to determine the calibration factors.

### 4.2. Pathology Detection

The implemented software was used to monitor the body surface temperature and the HR of the neonatal phantom. Therefore, the set values for both vital signs were adapted during the camera recordings. While the IRT frames were used to extract the surface temperature of different body parts to measure pathological thermoregulatory process and further the cpTD, the RGB images were analyzed for the PPGI signal to derive the simulated HR.

**Figure 9:**
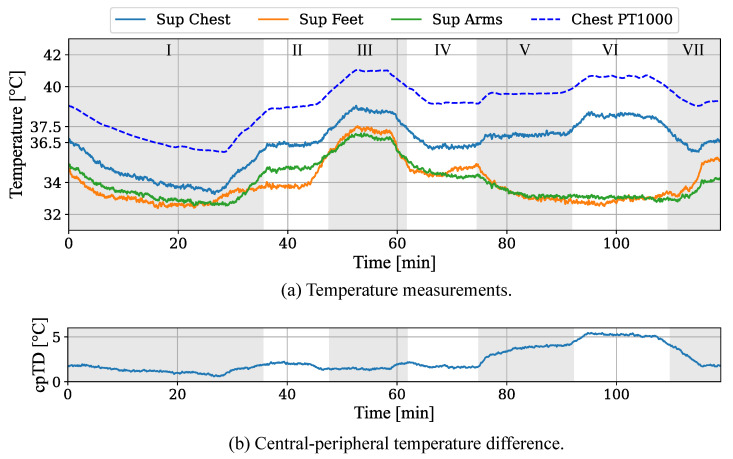
(**a**) Results for temperature measurements of camera-based setup and internal Pt1000 sensor. (**b**) Resulting central-peripheral temperature difference.

An illustrative example was chosen. The results for the temperature simulation and camera-based measurements are illustrated in Figure 9a. The set temperatures for different body parts were changed during a 120-min simulation to enable pathological cpTDs. The resulting temperature values for central and peripheral body areas are plotted next to the reference temperature from the Pt1000 chest temperature sensor. In Figure 9b the cpTDs are presented which were measured using the chest for central and the mean of all available arms, hands, legs and feet for peripheral temperature values. The camera-based measurements for the individual body regions differ from their corresponding internal Pt1000 temperature probes, which were integrated on the scaffold during the construction of the neonatal phantom. This temperature difference can also be observed for other body parts. This can be seen exemplary in Figure 9a for the chest area.

The recording can be divided into seven phases, which are listed in Table 5. The initial temperature progress showed a drop in chest temperature below 36.5 ${}^{\circ }C$, stabilizing at 34 ${}^{\circ }C$. While this simulated a mild/moderate hypothermia, an induced normothermia in phase II with a resulting fever (skin temperature above 37.5 ${}^{\circ }C$) in phase III were observed. In the following phase IV, the set temperature was again reduced to simulate a normotherm progress.

Until the end of phase IV, the cpTD was less than 2 ${}^{\circ }C$ (cf. Figure 9b). The next phases V and VII simulated a rising cpTD by slightly increasing the temperature of central body parts while simultaneously decreasing the set value for the peripheral areas. The progress of the measured cpTD can be observed in Figure 9b. While phase V showed a cpTD of 4 ${}^{\circ }C$ in the steady state after the cooling of arms and feet, phase VI revealed a cpTD of almost 6 ${}^{\circ }C$ due to an increase in central temperature. The final phase VII shows the return to the initial physiological temperature values.

**Figure 10:**
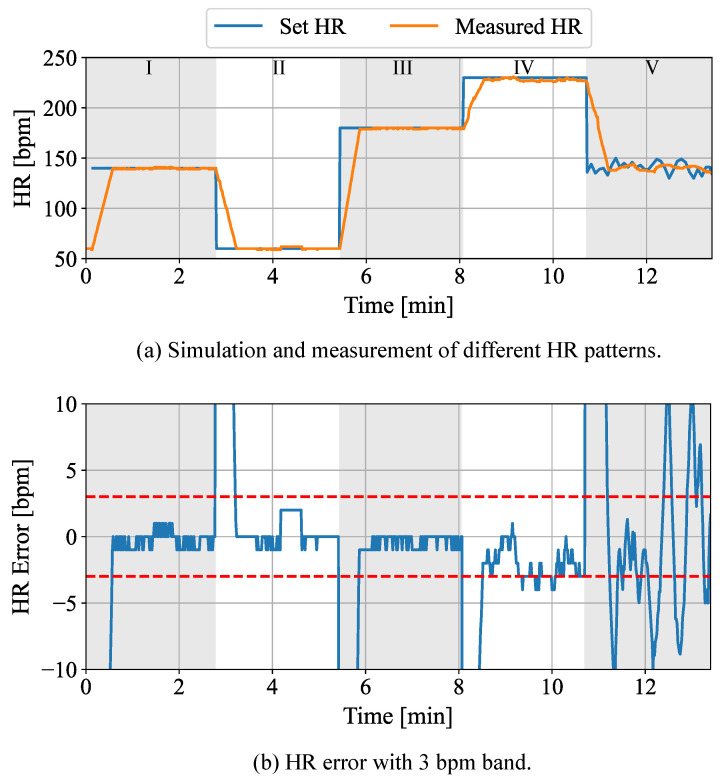
(**a**) Validation of HR detection and (**b**) the resulting measurement error. The dashed red lines in (**b**) show the 3 bpm band.

Next to the simulation of thermoregulatory conditions of the neonatal phantom, the capabilities of a cardiac-related pathology progression were evaluated using the PPG functionality. Thus, the set HR was modified during a recording, while simultaneously measuring the PPGI signal using the pathology detection software. In Figure 10a, the simulated patterns and the camera-based measurements of the HR are presented. Additionally, Figure 10b shows the resulting measurement error. In Table 6, the classification of the simulated phases is provided.

In the beginning of the simulation (phase I), a physiological HR of 140 bpm was set. Subsequently, the set HR was reduced to a value less than 60 bpm in order to prove the simulation of a severe bradycardia in phase II. In phases III and IV, the HR was increased to values near a tachycardia (180 bpm) and then to a severe tachycardia (230 bpm). Finally, the HR was changed back to a physiological (140 bpm) in phase V with a simulated HRV. In Figure 10b, the HR error can be observed. The dashed red lines describe the error margins of ±3 bpm. Since the HR was computed using a sliding-window approach with a window length of 30 s, the prediction error for the extracted HR regularly increased during transient phases. For phases I to IV, a mean absolute error of 0.89 bpm can be observed. In contrast, the mean absolute error increased to 4.62 bpm in phase V with a simulated HRV. The calculated errors only include the time periods, where the measured heart rate already reached the set point. As can be observed in Figure 10, the results show an accurate camera-based measurement of the HR in the phases using the implemented setup.

## 5. Discussion

### 5.1. Thermal Validation

The absolute temperature accuracy for the Leptons was originally stated as ±$5\;{}^{\circ }C$ in the data sheet [31] within the relevant temperature range of 30–$40\;{}^{\circ }C$. The measured maximum errors of the Leptons of up to $8\;{}^{\circ }C$ (cf. Figure 8) even exceeded the nominal error, most likely because of the absorption of the IR window, which favors underestimation. As demonstrated in this paper (cf. Table 4), proper calibrations of the Leptons with an active thermal reference can lead to an accuracy improvement of at least 79% ($8\;{}^{\circ }C$ to $1.64\;{}^{\circ }C$).

Normally, one would expect that the error is smallest in the measurement that was used to determine calibration factors. However, the calibrated temperature of the validation measurement was found to have a higher accuracy. In contrast to the validation measurement, the temperature data used to determine the calibration showed a thermal hysteresis for the thermal camera. The Leptons measured different temperatures, although the active reference body had the same temperature. One reason could be the different thermal behavior during heating or cooling of the reference body. However, since this behavior was not seen in the validation measurement, a quantitative analysis should verify this in the future.

Additionally, the custom-made active reference body and its usage impose a potential error during the measurements. The reference did not have an absolute homogeneous temperature distribution, especially during heat-up, and the skin temperature probe was isolated and contacted by thermal contact fluid. In future studies, a passive reference inside the FOV could be used to perform a Live-Offset compensation. In this case, not only a one-time calibration was conducted to compensate the infrared window, but an offset compensation was performed continuously during the measurements. Finally, research should be conducted concerning the temperature difference between the core and the skin temperature.

### 5.2. Pathology Detection

The results of the temperature measurements demonstrated the feasibility to simulate thermoregulatory processes and the capability of a camera-based detection of pathological conditions. While the camera-based temperatures measured from the skin showed an accurate relative behavior for the chest area, the absolute values were underestimated, with a mean temperature difference of approx. $2.50\;{}^{\circ }C$. This can be explained with the position of the Pt1000 sensor, which was located inside the neonatal phantom next to the heating elements, whereas the Lepton recorded lower temperatures from the outer skin. Further, the emissivity of the colored silicone could influence the remote temperature measurement. In future work, the integration of the Pt1000 sensors into the silicone layer needs to be investigated. Additionally, a profound emissivity analysis of the silicone will be conducted.

The analysis of the HR simulation of the neonatal phantom and the monitoring using the implemented setup demonstrated that the developed algorithms enabled the real-time extraction of a PPGI signal. Several pathological HR levels between 60 and 230 bpm were simulated. The analysis revealed a MAE of 0.89 bpm during the phases described in Section 4.2. The resulted high accuracy can be explained by the reduction of potential external impacts for the PPGI measurement. However, the simulation of a physiological HRV led to errors of up to 4.62 bpm. In this case, the sliding window approach with a window length of 30 s prevented a higher accuracy.

Unlike real infants, the phantom was lying still inside the incubator without any movement, which describes one of the biggest problems in camera-based vital signs measurement. In future work, a movement simulation will be added to the neonatal phantom using actuators. Further, the dependencies of PPGI measurements on ambient conditions will be analyzed.

## 6. Conclusions

During this work, a neonatal phantom was used to simulate various stages of hypothermia and hyperthermia. In addition, cardiac-related pathologies, such as tachycardia and bradycardia, were simulated via LED modulation. The ability of the neonatal phantom to create a temperature distribution and a PPG signal in a controlled manner enabled the development and the validation of a contactless, camera-based monitoring system to measure the simulated vital signs and classify the pathologies. The accuracy of the temperature measurements was improved by at least 79% with a maximum absolute error of less than $1.64\;{}^{\circ }C$ by using an active thermal reference body, compensating the spectral absorption of the HR window. A DL-based pre-trained human pose estimator was used to determine the ROIs of the hands, the feet, the chest and the head, allowing to semantically match the measured skin temperatures with the areas of the body. Further, the PPGI signal was extracted, allowing to supervise pathological HR conditions.

Even though the presented system achieved promising measurement accuracies and provided a reliable detection of pathologic states, challenges remain, which will be addressed in future work. Since the error for temperature measurement is still relatively high with a maximum error of $1.64\;{}^{\circ }C$, it would be feasible to add a passive thermal reference for live offset compensation or use a data-driven approach for temperature correction. Furthermore, the functionality of PPGI recordings could be extended by adding another camera with an IR filter for measuring the oxygen saturation, which can be simulated by the neonatal phantom already. An additional series of measurements should analyze the emissivity of the phantom’s skin to evaluate the impact of the ambient temperature, as well as measurements involving a more realistic temperature and humidity inside the incubator. Finally, the integration of motors into the neonatal phantom to enable the simulation of motion artifacts will be addressed in the future.

## Figures and Tables

**Table 1:** Simulated temperature-dependent pathologies.

Pathology	Feature Threshold Value
Mild Hypothermia	$36\;{}^{\circ }C\leq T<36.5\;{}^{\circ }C$
Moderate Hypothermia	$32\;{}^{\circ }C\leq T<36\;{}^{\circ }C$
Severe Hypothermia	$T<32\;{}^{\circ }C$
Hyperthermia	$37.5\;{}^{\circ }C<T<40\;{}^{\circ }C$
Severe Hyperthermia	$T\geq 40\;{}^{\circ }C$
Increased cpTD	$|\Delta T|>2\;{}^{\circ }C$

**Table 2:** Simulated HR-dependent pathologies.

Pathology	HR Threshold Value
Tachycardia	>200 bpm
Severe Tachycardia	>220 bpm
Bradycardia	<100 bpm
Severe Bradycardia	<80 bpm

**Table 3:** Camera properties and parameters used in this work.

Property	Arducam Mini HQ [30]	Lepton 3.5 [31]
Spectral Range	Visual	LWIR
Resolution [px]	1920 × 1080	160 × 120
Framerate [Hz]	30	8.7
Output Format [bit]	Raw: 10	Raw: 14
HFOV [${}^{\circ }$]	75	57

**Table 4:** Accuracy of the calibrated fitting and validation measurement.

	Lepton 1 (Fitting) (°C)	Lepton 2 (Validation) (°C)
MAE	0.55	0.24
STD	0.69	0.27
MAX	1.64	0.74
MIN	−1.15	−1.22

**Table 5:** Classification of temperature simulation from Figure 9.

Phase	Condition	cpTD > 2 ${}^{\circ }$C
I	Mild/Moderate Hypothermia	No
II	Normothermia	No
III	Hyperthermia	No
IV	Normothermia	No
V	Centralization	Yes
VI	Severe Centralization	Yes
VII	Normothermia	No

**Table 6:** Classification of HR simulation from Figure 10.

Phase	Condition
I	Physiological
II	Severe Bradycardia
III	Physiological
IV	Severe Tachycardia
V	Physiological with simulated HRV

## References

[1] UNICEF Data: United Nations Inter-Agency Group for Child Mortality Estimation (UN IGME) Levels and Trends in Child Mortality 2020. https://data.unicef.org/resources/levels-and-trends-in-child-mortality/.

[2] Bont L., Bernlöhr A., Abbott J., Mader S., Thiele N. (2012). Caring for Tomorrow: EFCNI White Paper on Maternal and Newborn Health and Aftercare Services. https://www.researchgate.net/publication/344632275_Caring_for_Tomorrow_EFCNI_White_Paper_on_Maternal_and_Newborn_Health_and_Aftercare_Services.

[3] World Health Organization (2020). Newborns: Improving Survival and Well-Being. https://www.who.int/news-room/fact-sheets/detail/newborns-reducing-mortality.

[4] World Health Organization (2018). Preterm Birth. https://www.who.int/news-room/fact-sheets/detail/preterm-birth.

[5] Lunze K., Bloom D.E., Jamison D.T., Hamer D.H. (2013). The global burden of neonatal hypothermia: Systematic review of a major challenge for newborn survival. BMC Med..

[6] Lyon A., Püschner P. (1995). Thermomonitoring: A Step Forward in Neonatal Intensive Care.

[7] Knobel R.B., Guenther B.D., Rice H.E. (2011). Thermoregulation and thermography in neonatal physiology and disease. Biol. Res. Nurs..

[8] Knobel R.B., Holditch-Davis D., Schwartz T.A., Wimmer J. (2009). Extremely low birth weight preterm infants lack vasomotor response in relationship to cold body temperatures at birth. J. Perinatol..

[9] Oranges T., Dini V., Romanelli M. (2015). Skin physiology of the neonate and infant: Clinical implications. Adv. Wound Care.

[10] Lam H., Kostov Y., Tolosa L., Falk S., Rao G. (2012). A high-resolution non-contact fluorescence-based temperature sensor for neonatal care. Meas. Sci. Technol..

[11] Morris A. (2020). No Wires, More Cuddles. https://news.northwestern.edu/stories/2019/02/wireless-body-sensors-premature-babies-nicu/.

[12] Antink C.H., Lyra S., Paul M., Yu X., Leonhardt S. (2019). A broader look: Camera-based vital sign estimation across the spectrum. Yearb. Med. Inform..

[13] Lyra S., Mayer L., Ou L., Chen D., Timms P., Tay A., Chan P.Y., Ganse B., Leonhardt S., Hoog Antink C. (2021). A Deep Learning-Based Camera Approach for Vital Sign Monitoring Using Thermography Images for ICU Patients. Sensors.

[14] Huang B., Chen W., Lin C.L., Juang C.F., Xing Y., Wang Y., Wang J. (2021). A neonatal dataset and benchmark for non-contact neonatal heart rate monitoring based on spatio-temporal neural networks. Eng. Appl. Artif. Intell..

[15] Cao Z., Simon T., Wei S.E., Sheikh Y. Realtime multi-person 2d pose estimation using part affinity fields. Proceedings of the IEEE Conference on Computer Vision and Pattern Recognition (CVPR).

[16] Ansari A.H., Cherian P.J., Caicedo A., Naulaers G., De Vos M., Van Huffel S. (2019). Neonatal Seizure Detection Using Deep Convolutional Neural Networks. Int. J. Neural Syst..

[17] Ihlen E.A.F., Støen R., Boswell L., de Regnier R.A., Fjørtoft T., Gaebler-Spira D., Labori C., Loennecken M.C., Msall M.E., Möinichen U.I. (2020). Machine Learning of Infant Spontaneous Movements for the Early Prediction of Cerebral Palsy: A Multi-Site Cohort Study. J. Clin. Med..

[18] Turova V., Sidorenko I., Eckardt L., Rieger-Fackeldey E., Felderhoff-Müser U., Alves-Pinto A., Lampe R. (2020). Machine learning models for identifying preterm infants at risk of cerebral hemorrhage. PLoS ONE.

[19] Nagy Á., Földesy P., Jánoki I., Terbe D., Siket M., Szabó M., Varga J., Zarándy Á. (2021). Continuous Camera-Based Premature-Infant Monitoring Algorithms for NICU. Appl. Sci..

[20] Villarroel M., Chaichulee S., Jorge J., Davis S., Green G., Arteta C., Zisserman A., McCormick K., Watkinson P., Tarassenko L. (2019). Non-contact physiological monitoring of preterm infants in the neonatal intensive care unit. NPJ Digit. Med..

[21] Khanam F.T.Z., Perera A.G., Al-Naji A., Gibson K., Chahl J. (2021). Non-contact automatic vital signs monitoring of infants in a neonatal intensive care unit based on neural networks. J. Imaging.

[22] Ervural S., Ceylan M. (2021). Classification of neonatal diseases with limited thermal Image data. Multimed. Tools Appl..

[23] Lyra S., Voss F., Coenen A., Blase D., Badiola Aguirregomezcorta I., Uguz D.U., Leonhardt S., Hoog Antink C. (2021). A Neonatal Phantom for Vital Signs Simulation. IEEE Trans. Biomed. Circuits Syst..

[24] Lubkowska A., Szymański S., Chudecka M. (2019). Surface body temperature of full-term healthy newborns immediately after Birth—Pilot study. Int. J. Environ. Res. Public Health.

[25] Zemlin M., Berger A., Franz A., Gille C., Härtel C., Helmut K., Müller A., Pohlandt F., Simon A., Merz W. (2019). Leitlinie der Gesellschaft für Neonatologie und Pädiatrische Intensivmedizin, der Deutschen Gesellschaft für Kinder- und Jugendmedizin, der Gesellschaft für Pädiatrische Gastroenterologie und Ernährung und der Deutschen Gesellschaft für Kinderchirurgie. AWMF Online, AWMF-Leitlinien-Register Nr. 024/008.

[26] McCormick M., Cooper P. (2003). Managing Newborn Problems: A Guide for Doctors, Nurses, and Midwives.

[27] World Health Organization (1997). Maternal and Newborn Health. Thermal Protection of the Newborn: A Practical Guide.

[28] American Academy of Pediatrics and the American College of Obstetricians and Gynecologists (2017). Guidelines for Perinatal Care.

[29] Gausepohl H.J., Pöschl J. (2020). Heidelberger Leidfaden Neonatalogie 2020.

[30] (2021). Arducam MINI High Quality Camera with M12 Mount Lens, 12.3MP 1/2.3 Inch IMX477 HQ Camera Module for Jetson Nano, Xavier NX. https://www.arducam.com/product/arducam-high-quality-camera-for-jetson-nano-and-xavier-nx-12mp-m12-mount/.

[31] Teledyne FLIR LLC (2021). LWIR Micro Thermal Camera Module 3 & 3.5. https://flir.netx.net/file/asset/15529/original/attachment.

[32] Jost K., Pramana I., Delgado-Eckert E., Kumar N., Datta A.N., Frey U., Schulzke S.M. (2017). Dynamics and complexity of body temperature in preterm infants nursed in incubators. PLoS ONE.

[33] Fischler M.A., Bolles R.C. (1981). Random sample consensus: A paradigm for model fitting with applications to image analysis and automated cartography. Commun. ACM.

[34] (2019). Real-Time Pose Estimation Accelerated with Nvidia Tensorrt. GitHub Repository. https://github.com/NVIDIA-AI-IOT/trt_pose.

[35] Xiao B., Wu H., Wei Y. Simple baselines for human pose estimation and tracking. Proceedings of the European Conference on Computer Vision (ECCV).

